# Gamma glutamyltransferase and risk of dementia in prediabetes and diabetes

**DOI:** 10.1038/s41598-020-63803-0

**Published:** 2020-04-22

**Authors:** Eugene Han, Ji-Yeon Lee, Kyung-do Han, Hanna Cho, Kwang Joon Kim, Byung-Wan Lee, Eun Seok Kang, Bong-Soo Cha, Zobair M Younossi, Yong-ho Lee

**Affiliations:** 1 Division of Endocrinology and Metabolism, Department of Internal Medicine, Keimyung University School of Medicine, Daegu, Korea; 2 Graduate School, Yonsei University College of Medicine, Seoul, Korea; 3 Division of Endocrinology and Metabolism, Department of Internal Medicine, Yonsei University College of Medicine, Seoul, Korea; 4 Department of Statistics and Actuarial Science, Soongsil University, Seoul, Korea; 5 Department of Neurology, Gangnam Severance Hospital, Yonsei University College of Medicine, Seoul, Korea; 6 Division of Geriatrics, Department of Internal Medicine, Yonsei University College of Medicine, Seould, Korea; 7 Center for Liver Diseases, Department of Medicine, Inova Fairfax Hospital, Galls Church, VA 22042 USA; 8 Intsitute of Endocrine Research, Yonsei University College of Medicine, Seoul, Korea; 9 Department of Systems Biology, Glycosylation Network Research Center, Yonsei University, Seoul, Korea

**Keywords:** Alzheimer's disease, Diabetes complications

## Abstract

Diabetes is associated with cognitive impairment and greater risk for dementia, but the role of gamma-glutamyltransferase (γ-GT) in dementia has not been elucidated. We determined incident dementia including Alzheimer’s disease and vascular dementia, analyzing data from participants aged 40 years or older in the National Health Insurance Database, collected by the National Health Insurance Service in Korea, from January 2009 to December 2015. During a median follow-up of 7.6 years, 272,657 participants were diagnosed as having dementia. Higher serum γ-GT was associated with increased risk of dementia (HR = 1.22, 95% CI = 1.20–1.24), and had a strong positive association with early onset dementia (HR = 1.32, 95% CI = 1.24–1.40). An additive impact of higher γ-GT on dementia was observed regardless of glycemic status, and prevalent diabetes with the highest γ-GT quartile had a 1.8-fold increased dementia risk (HR = 1.82, 95% CI = 1.78–1.85). This effect of γ-GT concentration in diabetes was more prominent in individuals with vascular dementia (HR = 1.94, 95% CI = 1.84–2.04). In subgroup analysis, young age, male sex, and relatively healthy subjects with a higher γ-GT quartile had more increased dementia risk. In conclusion, γ-GT concentration as well as glycemic status could be a future risk factor for dementia in the general population.

## Introduction

Over the past few decades, population aging has produced increased cases of dementia. Dementia is the leading cause of chronic disability in elderly individuals^1,2^. The worldwide prevalence of dementia was approximately 35.6 million in 2010, and the number of individuals living with dementia is expected to double every 20 years^3^. In addition, early onset dementia (EOD) can lead to significant socioeconomic burden and impaired quality of life in the younger population^4,5^, Along with genetic risk factors, inflammatory pathways and vascular factors have been implicated in the development of dementia. It is reported that chronic systemic inflammation could lead to cognitive dysfunction and substantially increase the risk of cognitive impairment^6^. Among chronic inflammatory diseases, diabetes is thought to play an important role in cognitive dysfunction^7^. Atherosclerosis is not only implicated in vascular dementia (VaD), but is also an independent risk factor for Alzheimer’s disease (AD), which suggests its importance in dementia regardless of subtype^8^.

Gamma-glutamyltransferase (γ-GT) is known as a marker of hepatobiliary disease^9^. γ-GT is a highly sensitive enzyme whose level may be elevated in liver disease such as non-alcoholic fatty liver disease (NAFLD). Recent studies demonstrate the possible role of γ-GT in systemic diseases; large population-based studies show the association between γ-GT and vascular disease^10,11^. Furthermore, correlations between γ-GT in inflammation^12^, and oxidation^13^ have been demonstrated. In this context, it is plausible that elevated γ-GT in liver disease shares some common mechanisms in dementia. We hypothesized that γ-GT may be independently associated with dementia. Therefore, the aim of this study was to investigate the impact of γ-GT in dementia and the association between γ-GT, diabetes, and dementia risk in a general population.

## Results

### Study population

**Figure 1:**
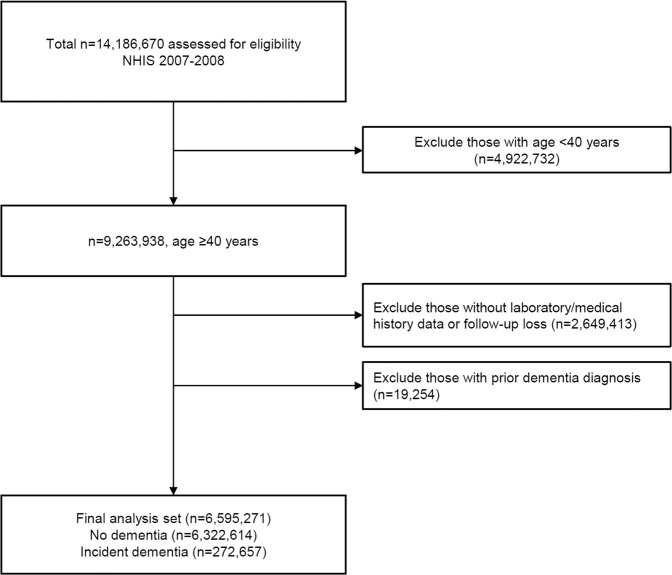
Study flow. NHIS, Korean National Health Insurance Service.

Among a study population of 6,595,271 adults, 272,657 had a new diagnosis of dementia during the follow-up period (Fig. 1). The mean duration of follow-up was 7.6 years, and the overall incidence of dementia during the entire study period was 4.1% (272,657/6,595,271), and AD in late oneset dementia (LOD) ranked the highest incidence (11.4%). The mean age of the study population was 55.9 years, and the mean age of dementia diagnosis was 76.5 years (Table 1). Individuals with AD were older than those with VaD, and participants with incident dementia had higher blood pressure, fasting plasma glucose, and total cholesterol at baseline compared to participants who remained dementia-free over follow-up (all P < 0.001). The proportion of hypertension, dyslipidemia, myocardial infarction, ischemic stroke, prevalent diabetes, and incident diabetes was higher among participants who developed dementia over follow-up. Regarding γ-GT quartiles, body mass index (BMI), blood pressure, total cholesterol, aspartate transaminase (AST), and alanine transferase (ALT) were increased from lowest quartile to highest quartile (Table 2). Glycemic status and fasting plasma glucose value was higher for greater quartile of γ-GT.

**Table 1:** Baseline characteristics of study population.

	Overall (N = 6,595,271)	Without dementia (N = 6,322,614)	With dementia (N = 272,657)	*P* value
Age (years) at survey, mean (SD)	55.9 (10.4)	55.2 (10.0)	71.7 (7.9)	<0.001
Age ≥60 years, N (%)	2,244,030 (34.0)	1,991,288 (31.5)	252,742 (92.7)	<0.001
Male, N (%)	2,467,983 (37.4)	2,386,726 (37.8)	81,257 (29.8)	<0.001
BMI (kg/m^2^), mean (SD)	23.9 (3.0)	23.9 (3.0)	23.7 (3.3)	<0.001
Obesity, N (%)*	2,199,321 (33.3)	211,0251 (33.4)	89,070 (32.7)	<0.001
SBP (mmHg), mean (SD)	124.5 (16.2)	124.2 (16.1)	131.2 (17.5)	<0.001
DBP (mmHg), mean (SD)	77.0 (10.3)	77.0 (10.3)	78.9 (10.5)	<0.001
FPG (mg/dL), mean (SD)	98.1 (24.6)	97.8 (24.2)	105.0 (32.5)	<0.001
Total cholesterol (mg/dL), mean (SD)	198.2 (37.0)	198.2 (36.9)	200.0 (39.7)	<0.001
γ-GT (IU/L), median (IQR)	24.0 (24.0–24.0)	24.1 (24.0–24.1)	23.8 (23.7–23.8)	<0.001
AST (IU/L), median (IQR)	24.4 (24.4–24.4)	24.4 (24.4–24.4)	25.2 (25.2–25.2)	<0.001
ALT (IU/L), median (IQR)	21.9 (21.9–21.9)	22.0 (22.0–22.0)	20.3 (20.3–20.4)	<0.001
Glycemic status				<0.001
Normoglycemia, N (%)	198,210 (3.0)	186,322 (3.0)	11,888 (4.4)	
IFG, N (%)	4,372,692 (66.3)	4,229,999 (66.9)	142,693 (52.3)	
Incident diabetes, N (%)	1,495,510 (23.7)	1,428,522 (22.6)	66,988 (24.6)	
Prevalent diabetes, N (%)	528,859 (8.0)	477,771 (7.6)	51,088 (18.7)	
Hypertension, N (%)	2,285,042 (34.6)	2,118,179 (33.5)	166,863 (61.2)	<0.001
Dyslipidemia, N (%)	1,394,308 (21.1)	1,313,660 (20.8)	80,648 (29.6)	<0.001
Myocardial infarction, N (%)	41,228 (0.6)	37,295 (0.6)	3,933 (1.4)	<0.001
Stroke, N (%)	178,144 (2.7)	146,557 (2.3)	31,587 (11.6)	<0.001
Current smoker, N (%)	722,293 (11.0)	700,470 (11.1)	21,823 (8.0)	<0.001
Heavy drinker, N (%)	1,204,882 (18.3)	1,185,234 (18.8)	19,648 (7.2)	<0.001
Regular exercise, N (%)	3,065,437 (46.5)	2,976,362 (47.1)	89,075 (32.7)	<0.001
Income, lowest quartile, N (%)	1,399,220 (21.2)	1,343,661 (21.3)	55,559 (20.4)	<0.001

Data for continuous variables were expressed as either mean ± standard deviation, or mean (interquartile range) and categorical variables were expressed as number (percent).

*Obesity was defined as body mass index >25 kg/m^2^ per Asian-Pacific definition. Abbreviations: BMI, body mass index; SBP, systolic blood pressure; DBP, diastolic blood pressure; FPG, fasting plasma glucose; γ-GT, gamma-glutamyltransferase; AST, aspartate transaminase; ALT, alanine transferase; IFG, impaired fasting glucose.

**Table 2:** Sex-specific γ-GT and metabolic parameters.

	γ-GT Q1 (lowest) (N = 1,667,971)	γ-GT Q2 (N = 1,625,459)	γ-GT Q3 (N = 1,702,051)	γ-GT Q4 (highest) (N = 1,599,790)	*P* value
γ-GT (IU/L), median (IQR)	12.0 (10.0–14.0)	17.0 (15.0–23.0)	23.0 (20.0–32.0)	46.0 (32.0–67.0)	<0.001
γ-GT (IU/L), cutoff in men	≤19.0	20.0–28.0	29.0–43.0	≥44.0	<0.001
γ-GT (IU/L), cutoff in women	≤13.0	14.0–17.0	18.0–25.0	≥26.0	<0.001
Age (years) at survey, mean (SD)	55.0 (10.8)	55.8(10.5)	56.4(10.3)	56.3(9.9)	<0.001
Age ≥60 years, N (%)	522,568 (31.3)	551,870 (34.0)	610,178 (35.9)	559,414 (35.0)	<0.001
Follow-up duration (years), mean (SD)	7.7 (1.2)	7.7 (1.2)	7.6 (1.2)	7.6 (1.3)	<0.001
Men, N (%)	575,297 (34.5)	675,673 (41.6)	606,913 (35.7)	610,100 (38.1)	<0.001
Dementia, N (%)	64,217 (3.9)	64,442 (4.0)	73458 (4.3)	70540 (4.4)	<0.001
BMI (kg/m^2^), mean (SD)	22.9 (2.7)	23.6 (2.8)	24.3 (3.0)	24.9 (3.2)	<0.001
Obesity, N (%)*	331,383 (20)	467,720 (29)	647,821 (38)	752,397 (47)	<0.001
SBP (mmHg), mean (SD)	121.3 (15.9)	123.6 (15.9)	125.5 (16.1)	127.5 (16.3)	<0.001
DBP (mmHg), mean (SD)	75.0 (10.1)	76.6 (10.1)	77.7 (10.2)	79.0 (10.4)	<0.001
FPG (mg/dL), mean (SD)	93.6 (18.8)	96.1 (21.8)	98.8 (24.8)	104.1 (30.5)	<0.001
Total cholesterol (mg/dL), mean (SD)	188.2 (33.7)	196.0 (35.0)	202.1 (36.9)	206.9 (39.8)	<0.001
AST (U/L), median (IQR)	21.8 (21.8–21.8)	23.0 (23.0–23.0)	24.3 (24.3–24.3)	29.3 (29.3–29.3)	<0.001
ALT (U/L), median (IQR)	17.1 (17.1–17.1)	19.7 (19.7–19.7)	22.5 (22.5–22.5)	30.7 (30.7–30.7)	<0.001
Glycemic status					
Normoglycemia, N (%)	1,258,644 (75.5)	1,136,977 (67.0)	1,093,089 (64.2)	883,982 (55.3)	<0.001
IFG, N (%)	303,121 (18.2)	347,155 (21.4)	410,325 (24.1)	434,909 (27.2)	<0.001
Incident diabetes, N (%)	25,597 (1.5)	35,758 (2.2)	52,362 (3.1)	84,493 (5.3)	<0.001
Prevalent diabetes, N (%)	80,609 (4.8)	105,569 (6.5)	146,275 (8.6)	196,406 (12.3)	<0.001
Hypertension, N (%)	418,596 (25.1)	512,414 (31.5)	643,843 (37.8)	710,189 (44.4)	<0.001
Dyslipidemia, N (%)	199,912 (12.0)	286,018 (17.6)	409,593 (24.1)	498,785 (31.2)	<0.001
Myocardial infarction, N (%)	7,896 (0.5)	9,711 (0.6)	11,283 (0.7)	12,338 (0.8)	<0.001
Stroke, N (%)	34,742 (2.1)	40,853 (2.5)	49,595 (2.9)	52,954 (3.3)	<0.001
Current smoker, N (%)	151,403 (9.1)	192,585 (11.9)	188,063 (11.1)	190,242 (11.9)	<0.001
Heavy drinker, N (%)	260,994 (15.7)	298,898 (18.4)	317,239 (18.6)	327,751 (20.5)	<0.001
Regular exercise, N (%)	794,907 (47.7)	779,254 (47.9)	785,860 (46.2)	705,416 (44.1)	<0.001
Income, lowest quartile, No. (%)	357,888 (21.5)	340,007 (20.9)	360,958 (21.2)	340,367 (21.3)	<0.001

Data for continuous variables were expressed as either mean ± standard deviation, or mean (interquartile range) and categorical variables were expressed as number (percent). Abbreviations: BMI, body mass index; SBP, systolic blood pressure; DBP, diastolic blood pressure; FPG, fasting plasma glucose; γ-GT, gamma-glutamyltransferase; AST, aspartate transaminase; ALT, alanine transferase; IFG, impaired fasting glucose.

*Obesity was defined as body mass index >25 kg/m^2^ per Asian-Pacific definition.

### Incidence and risk for dementia according to serum γ-GT levels

**Figure 2:**
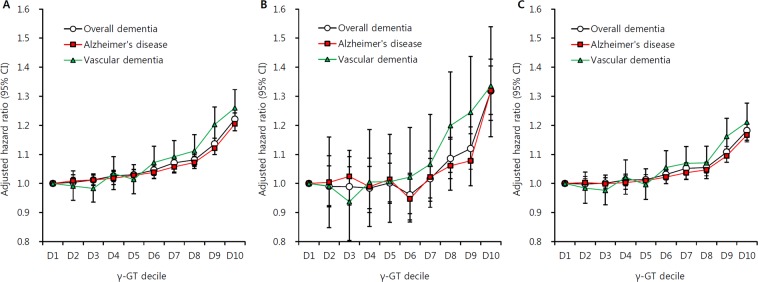
Gamma glutamyltransferase increases the risk of dementia. Risk for (**A**) overall dementia, (**B**) early onset dementia (age 40–59 years), and (**C**) late onset dementia (age ≥60 years) according to γ-GT decile. Adjusted for age, sex, body mass index, smoking history, alcohol use, regular exercise, hypertension, diabetes, dyslipidemia, myocardial infarction, ischemic stroke, and socioeconomic status.

**Figure 3:**
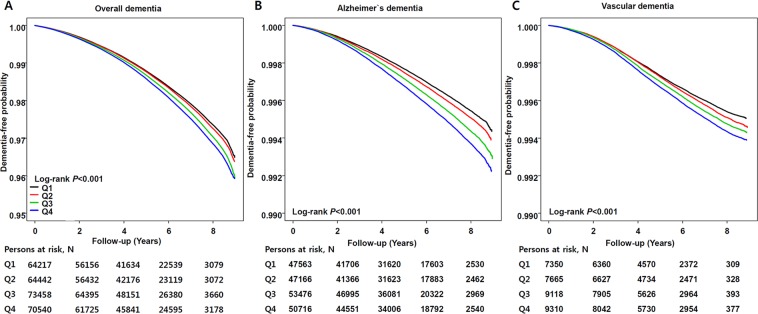
Dementia free survival determination by Kaplan-Meier curve according to γ-GT quartiles. (**A**) Overall dementia, (**B**) Alzheimier’s disease, and (**C**) vascular dementa. Lowest γ-GT quartile (black solid), 2nd γ-GT quartile (red solid), 3rd γ-GT quartile (green solid), and highest γ-GT quartile (blue solid).

To explore the relationship between baseline γ-GT and dementia risk, we categorized by sex-specific γ-GT decile and analyzed hazard ratios (HRs) for each group. Dementia risk increased gradually from the lowest to highest γ-GT decile (HR = 1.22, 95% confidence interval [CI] = 1.20–1.24 for the highest γ-GT quartile; Fig. 2). Among γ-GT quartiles, dementia incidence tended to increase from lowest to highest γ-GT quartiles (3.9 vs. 4.0 vs. 4.3 vs. 4.4, P < 0.001; Table 2). This result was observed regardless of age group; however, a much stronger association between dementia risk and γ-GT concentration was shown in EOD (HR = 1.32, 95% CI = 1.24–1.40) compared to LOD (HR = 1.18, 95% CI = 1.16–1.20). Regarding dementia subtype, the increasing risk pattern according to γ-GT concentration for AD (HR = 1.21, 95% CI = 1.18–1.23) was comparable to VaD (HR = 1.26, 95% CI = 1.20–1.32). The dementia-free survival curve also demonstrated that higher γ-GT quartile was associated with incident dementia regardless of subtype (Fig. 3). In the Cox analysis model, the highest γ-GT quartile was associated with higher overall dementia incidence by 16% compared to the lowest γ-GT group (HR = 1.16; 95% CI = 1.15–1.17, Table 3). Although incidence of LOD was higher than that of EOD, the risk for individuals with higher γ-GT values was more pronounced for EOD (HR = 1.22, 95% CI = 1.17–1.27) compared to LOD (HR = 1.13, 95% CI = 1.12–1.14). Similar patterns were observed for incidence of AD (HR = 1.14, 95% CI = 1.13–1.16) and VaD (HR = 1.22, 95% CI = 1.18–1.26). Individuals with NAFLD had increased risk for overall dementia types, particularly vascular dementia (HR = 1.11, 95% CI = 1.08–1.15).

**Table 3:** Cox model for dementia by γ-GT quartiles and NAFLD.

		Incident cases	Person-years	Incident rate*	HR (95% CI)
Overall dementia	γ-GT Q1	64,217	12,804,671	5.01	1 (Referent)
	γ-GT Q2	64,442	12,457,297	5.17	1.02 (1.01–1.03)
	γ-GT Q3	73,458	13,014,700	5.64	1.06 (1.05–1.07)
	γ-GT Q4	70,540	12,130,175	5.81	1.16 (1.15–1.17)
	NAFLD(−)	215,192	38,529,781	5.59	1 (Referent)
	NAFLD(+)	57,465	11,877,062	4.84	1.08 (1.07–1.09)
Early onset dementia	γ-GT Q1	4,117	8,999,668	0.46	1 (Referent)
	γ-GT Q2	4,385	8,417,327	0.52	1.00 (0.96–1.04)
	γ-GT Q3	5,066	8,542,570	0.59	1.01 (0.97–1.06)
	γ-GT Q4	6,347	8,086,032	0.78	1.22 (1.17–1.27)
	NAFLD(−)	14,402	26,077,278	0.55	1 (Referent)
	NAFLD(+)	5,513	7,968,320	0.69	1.02 (0.98–1.06)
Late onset dementia	γ-GT Q1	60,100	3,805,003	15.80	1 (Referent)
	γ-GT Q2	60,057	4,039,970	14.87	1.01 (1.00–1.02)
	γ-GT Q3	68,392	4,472,130	15.29	1.05 (1.03–1.06)
	γ-GT Q4	64,193	4,044,142	15.87	1.13 (1.12–1.14)
	NAFLD(−)	200,790	12,452,504	16.12	1 (Referent)
	NAFLD(+)	51,952	3,908,742	13.29	1.07 (1.05–1.08)
Alzheimer’s disease	γ-GT Q1	47,563	12,804,671	3.71	1 (Referent)
	γ-GT Q2	47,166	12,457,297	3.79	1.02 (1.00–1.03)
	γ-GT Q3	53,476	13,014,700	4.11	1.05 (1.04–1.06)
	γ-GT Q4	50,716	12,130,175	4.18	1.14 (1.13–1.16)
	NAFLD(−)	157,748	38,529,781	4.09	1 (Referent)
	NAFLD(+)	41,173	11,877,062	3.47	1.07 (1.06–1.09)
Vascular dementia	γ-GT Q1	7,350	12,804,671	0.57	1 (Referent)
	γ-GT Q2	7,665	12,457,297	0.62	1.02 (0.99–1.05)
	γ-GT Q3	9,118	13,014,700	0.70	1.10 (1.06–1.13)
	γ-GT Q4	9,310	12,130,175	0.77	1.22 (1.18–1.26)
	NAFLD(−)	25,548	38,529,781	0.66	1 (Referent)
	NAFLD(+)	7,895	11,877,062	0.66	1.11 (1.08–1.15)

Abbreviation: γ-GT, gamma-glutamyl transferase; NAFLD, nonalcoholic fatty liver disease; HR, hazard ratio; 95% CI, 95% confidence interval.

Adjusted for age, sex, body mass index, smoking status, alcohol use, exercise, hypertension, diabetes, dyslipidemia, myocardial infarction, stroke, and socioeconomic status.

*dementia incidence rate was expressed per 1000 person-years.

### Risk for dementia by glycemic status

**Figure 4:**
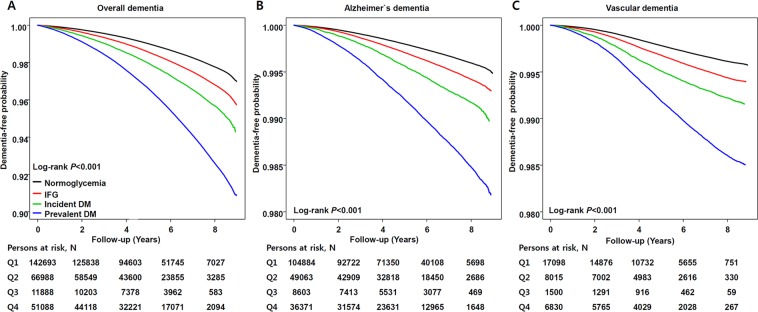
Dementia free survival determination by Kaplan-Meier curve according to glycemic status. (**A**) Overall dementia, (**B**) Alzheimier’s disease, and (**C)** vascular dementa. Normoglycemia (black solid), impaired fasting glucose (red solid), incident diabetes (green solid), and prevalent diabetes (blue solid).

The incidence of dementia was higher as glycemic status was increased (Table 4). Individuals with prevalent diabetes had a 1.6-fold greater risk for dementia compared to those with normal glucose concentration (HR = 1.60, 95% CI = 1.58–1.62) and this phenomenon was more prominent in EOD (HR = 1.73, 95% CI = 1.66–1.81) compared to LOD (HR = 1.55, 95% CI = 1.53–1.57). The risk for VaD (HR = 1.67, 95% CI = 1.62–1.72) was slightly higher compared to that of AD (HR = 1.55, 95% CI = 1.55–1.59). The difference in dementia risk according to glycemic status was also observed in the Kaplan-Meier curve and a similar pattern was shown in both AD and VaD (Fig. 4). As shown in Table 5, the risk for dementia ranked the highest in the treatment failure group. Subjects who fail to treat diabetes had a 2.07-fold greater risk for EOD.

**Table 4:** Cox model for dementia by glycemic status.

		Incident cases	Person-years	Incident rate*	HR (95% CI)
Overall dementia	Normoglycemia	142,693	33,676,334	4.24	1 (Referent)
	IFG	66,988	11,396,989	5.88	1.06 (1.05; 1.07)
	Incident diabetes	11,888	1,481,240	8.03	1.20 (1.18;1.22)
	Prevalent diabetes	51,088	3,852,280	13.26	1.60 (1.58; 1.62)
Early onset dementia	Normoglycemia	12,207	24,168,225	0.51	1 (Referent)
	IFG	4,421	7,324,881	0.60	1.04 (1.00; 1.07)
	Incident diabetes	680	899,137	0.76	1.21 (1.12; 1.30)
	Prevalent diabetes	2,607	1,653,354	1.58	1.73 (1.66; 1.81)
Late onset dementia	Normoglycemia	130,486	9,508,109	13.72	1 (Referent)
	IFG	62,567	4,072,107	15.36	1.06 (1.05; 1.07)
	Incident diabetes	11,208	582,103	19.25	1.22 (1.20; 1.24)
	Prevalent diabetes	48,481	2,198,926	22.05	1.55 (1.53; 1.57)
Alzheimer’s disease	Normoglycemia	104,884	33,676,334	3.11	1 (Referent)
	IFG	49,063	11,396,989	4.30	1.06 (1.05; 1.07)
	Incident diabetes	8,603	1,481,240	5.81	1.18 (1.15; 1.20)
	Prevalent diabetes	36,371	3,852,280	9.44	1.57 (1.55; 1.59)
Vascular dementia	Normoglycemia	17,098	33,676,334	0.51	1 (Referent)
	IFG	8,015	11,396,989	0.70	1.06 (1.03; 1.09)
	Incident diabetes	1,500	1,481,240	1.01	1.27 (1.21; 1.34)
	Prevalent diabetes	6,830	3,852,280	1.77	1.67 (1.62; 1.72)

Abbreviation: IFG, impaired fasting glucose; HR, hazard ratio; 95% CI, 95% confidence interval.

Adjusted for age, sex, body mass index, smoking status, alcohol use, exercise, hypertension, diabetes, dyslipidemia, myocardial infarction, stroke, and socioeconomic status.

*Dementia incidence rate was expressed per 1000 person-years.

**Table 5:** Risk for dementia by diabetes treatment status.

	Overall dementia	Early onset dementia	Late onset dementia	Alzheimer’s disease	Vascular dementia
Normoglycemia	1 (referent)	1 (referent)	1 (referent)	1 (referent)	1 (referent)
IFG	1.06 (1.05–1.07)	1.04 (1.01–1.07)	1.06 (1.05–1.07)	1.06 (1.05–1.07)	1.06 (1.03–1.09)
Incident diabetes	1.20 (1.18–1.22)	1.21 (1.12–1.30)	1.22 (1.20–1.24)	1.18 (1.15–1.20)	1.27 (1.21–1.34)
Treatment maintenance	1.59 (1.57–1.60)	1.70 (1.62–1.78)	1.54 (1.52–1.55)	1.56 (1.54–1.58)	1.65 (1.60–1.70)
Treatment failure	1.71 (1.66–1.76)	2.07 (1.83–2.34)	1.68 (1.64–1.73)	1.63 (1.58–1.68)	1.86 (1.73–2.01)
P value	<0.0001	<0.0001	<0.0001	<0.0001	<0.0001

Data were expressed as hazard ratio (95% confidence interval).

Abbreviation: IFG, impaired fasting glucose.

Adjusted for age, sex, body mass index, smoking status, alcohol use, exercise, hypertension, diabetes, dyslipidemia, myocardial infarction, stroke, and socioeconomic status.

### Combined effects of *γ-GT* levels and glycemic status on dementia risk

**Figure 5:**
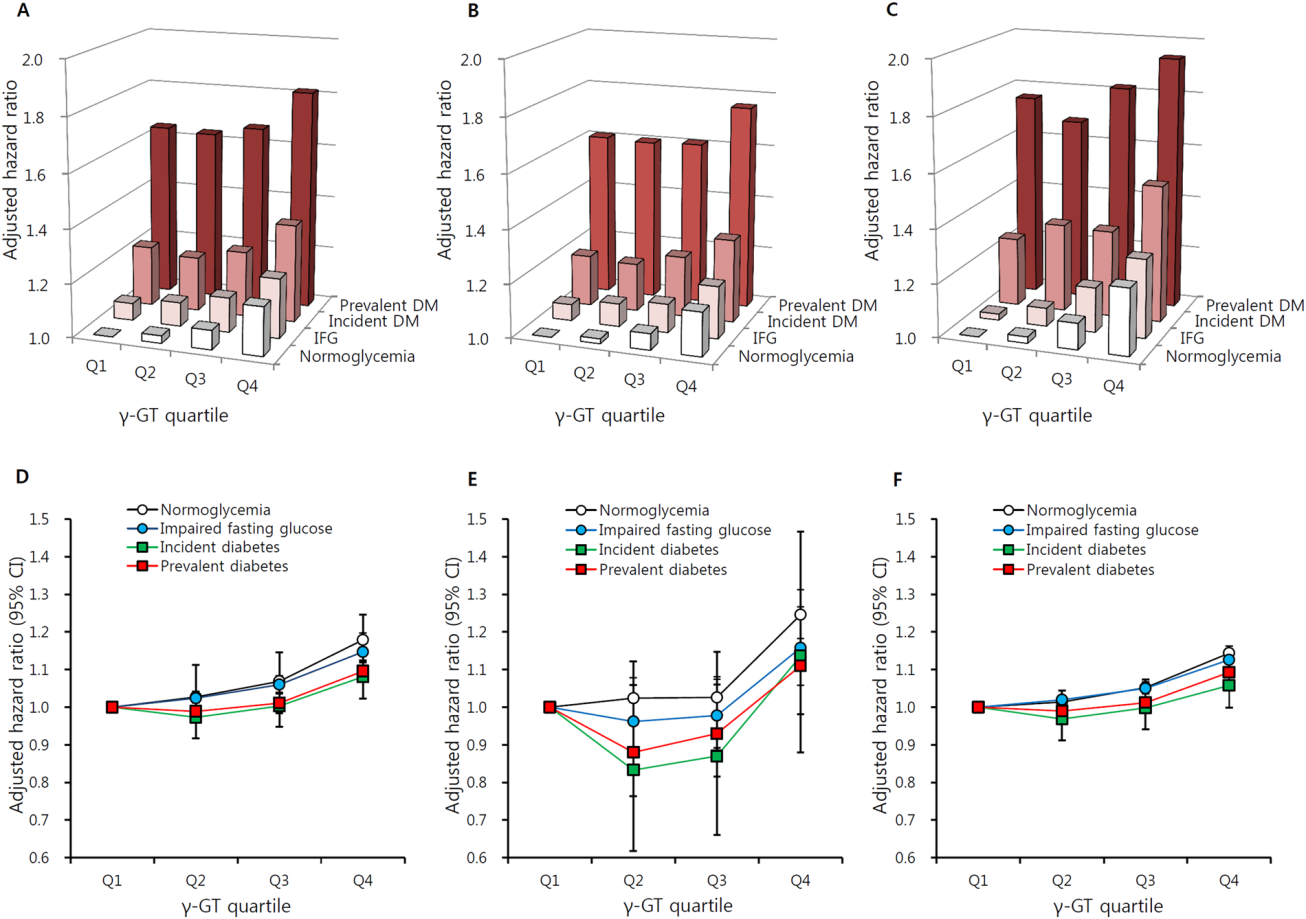
Hazard ratios for dementia according to γ-GT quartiles and glycemic status. Risk for (**A**) overall dementia, (**B**) Alzheimer’s disease, (**C**) vascular dementia. (**A**-**C**: normoglycemic with lowest γ-GT quartile as referent), (**D**) overall dementia, (**E**) early onset dementia (age 40–59 years), and (**F**) late onset dementia (age ≥60 years) (D-F: lowest γ-GT quartile in each glycemic status as referent). Adjusted for age, sex, body mass index, smoking history, alcohol use, regular exercise, hypertension, dyslipidemia, myocardial infarction, ischemic stroke, and income.

Compared to non-diabetic individuals with the lowest γ-GT concentration, those with prevalent diabetes and highest γ-GT concentrations had an approximately 1.8-fold increased risk of dementia (HR = 1.82, 95% CI = 1.78–1.85; Fig. 5A). This additive effect of γ-GT concentration in diabetes was more prominent in VaD (HR = 1.94, 95% CI = 1.84–2.04; Fig. 5B) than AD (HR = 1.76, 95% CI = 1.72–1.80; Fig. 5C). When analyzing the risk of dementia according to γ-GT quartiles among individuals with the same glycemic status (lowest γ-GT quartile as reference in each glycemic status group), the elevated hazard ratio for overall dementia was the most prominent for normoglycemia (HR = 1.18, 95% CI = 1.16–1.20; Fig. 5D) and prevalent diabetes and this pattern was similar in both EOD and LOD. The highest γ-GT quartile had was associated higher dementia risk regardless of glycemic status. In prevalent diabetes and incident diabetes in the younger age group, dementia risk was slightly lower in the second and third γ-GT quartiles and then increased in the highest quartile, representing a J shape (Fig. 5E). The risk for LOD in normoglycemic individuals in the highest γ-GT quartile was lower than EOD risk (HR = 1.14, 95% CI = 1.13–1.16; Fig. 5F).

### Association between *γ-GT* levels and the risk of dementia by other comorbid conditions

**Figure 6:**
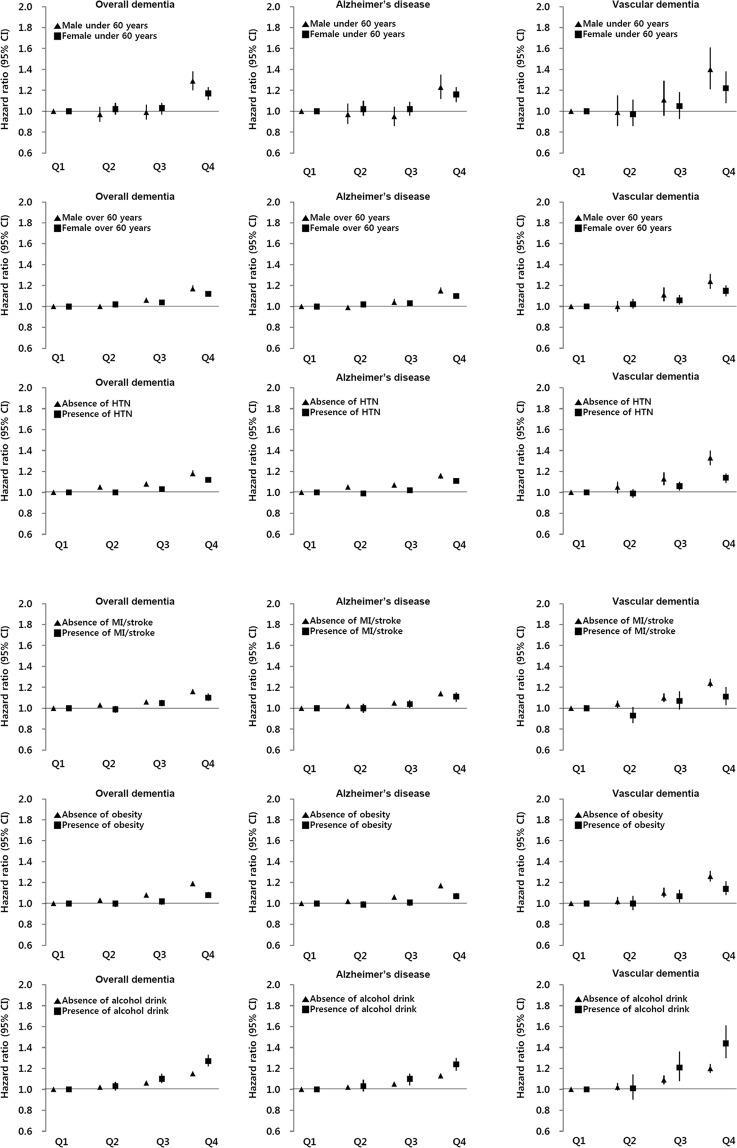
Hazard ratios and 95% confidence intervals for dementia incidence according to γ-GT quartile by gender, age, alcohol use, and comorbidities.

The association between γ-GT level and risk of dementia was still significant regardless of other clinical conditions (Fig. 6). The risk for highest γ-GT quartile in EOD was higher than in LOD for both sexes, and male individuals under 60 years old were had the greatest dementia risk, particularly for VaD (HR = 1.40, 95% CI = 1.21–1.61). VaD risk with higher γ-GT values was greater than AD risk regardless of hypertension, cardiovascular disease, and obesity. Regardless of dementia subtype, the impact of γ-GT on incident dementia was stronger in individuals without other comorbidities. In terms of alcohol consumption, heavy alcohol use with a higher γ-GT value had an increased dementia risk compared to individuals without heavy alcohol use, and VaD risk was the highest in heavy alcohol use group (HR = 1.44, 95% CI = 1.30–1.61).

## Discussion

In this large, national population-based study, we demonstrated that serum γ-GT levels had a linear relationship with risk of developing dementia, independent of glycemic status and other important potential confounders. In particular, the influences of higher γ-GT levels and diabetes on developing dementia were much stronger for EOD than for LOD. γ-GT concentrations and glycemic status had an additive impact on dementia incidence. Regarding glycemic categories, the association between higher serum γ-GT concentration and dementia risk was more profound in the normoglycemic group compared with impaired fasting glucose (IFG) or diabetes. In addition, the increased risk of developing VaD according to γ-GT concentration was greater compared to AD risk.

The impact of diabetes on dementia has been established, since other studies demonstrated chronic exposure to hyperglycemia may increase cognitive dysfunction^14,15^. Although there has been no study on the diabetes increases the risk of EOD, the evidence that average for dementia translation was earlier in patients with type 2 diabetes compared to the patients without diabetes^16^ accords our result. The remarkably increased EOD risk for treatment failure group might reflect that failure to treat diabetes may accelerate progression to cognitive decline and the importance of glucose control in young adults. Even among individuals without diabetes, the risk for future dementia gradually increased with elevated glucose level, and a lower glucose concentration was inversely associated with dementia risk^14^. In addition, even among individuals without diabetes, dementia risk increased as the glycemic status worsened, to IFG or incident diabetes. Notably, the association of γ-GT levels and dementia outcome was linear in the normoglycemic group in the current study. As to glycemic status, higher γ-GT concentrations in individuals with normal glucose levels had the highest dementia risk. As diabetes and dementia share common features^15^, and diabetes is often co-morbid with dementia^17^, the influence of elevated GGT in the normoglycemic group may be more pronounced.

Interestingly, in our study a strong positive association between serum γ-GT levels and dementia risk was observed in the younger age group, particularly in the young male, non-obese group. This is similar to previous studies of γ-GT and cardiovascular disease risk^18,19^. However, the association between γ-GT and dementia is independent of cardiovascular disease, as a stronger association was observed in individuals without previous myocardial infarction or ischemic stroke. Moreover, considering the smaller effect size of diabetes on EOD than LOD^17^, the degree of impact of γ-GT might be relatively greater on EOD than LOD. γ-GT levels are known to reflect vascular and cardiometabolic diseases and γ-GT enzyme activity is independently associated with cardiovascular disease and cardiovascular-related mortality, in a dose-dependent manner^10^, suggesting its causal relationship to vascular damage. In pooled analysis, higher γ-GT enzyme activity was a surrogate marker for diabetes and metabolic syndrome, which contribute to dementia pathogenesis^20^. Likewise, our study showed a relatively prominent impact of γ-GT on VaD compared to AD and a linear association between γ-GT quartiles and impaired glucose status. The pathways involved in γ-GT expression and cardiometabolic disease could be explained by insulin signaling impairment and insulin resistance^21^. Furthermore, γ-GT enzyme activity increased in all-cause and cancer mortality independently of NAFLD^18^, suggesting its essential role in human metabolism. γ-GT is primarily involved in extracellular catabolism of glutathione, the major thiol antioxidant that plays a protective role against oxidants^22^. Therefore, an increased serum γ-GT level reflects systemic oxidation and reactive oxygen species (ROS). We found more increased and stronger dementia risk according to γ-GT groups than NAFLD presence. In addition, one notable finding in our study was the J shape for dementia risk in the diabetes group. Individuals with diabetes have fundamentally increased ROS, and more γ-GT might be needed to compensate for this stress. If genetically or environmentally γ-GT concentration is decreased, the role of γ-GT in this defense mechanism would be impaired, with greater vulnerability to those conditions.

We acknowledge some limitations in the current study. First, this study was based on a national general health care dataset, which did not survey genetic factors such as *APOE* genotype. Second, we could not determine the dietary and lifestyle factors which could affect serum γ-GT concentration. However, we did adjust for alcohol consumption, which is a generally well-established γ-GT level determinant. Third, our analyses focused on dementia type based on diagnosis code rather than imaging or functional cognitive testing. Despite these limitations, our study has several strengths. First, as a large, population-based study, we achieved statistical reliability without selection bias. We were able to categorize into sex-specific γ-GT deciles or quintiles, while previous studies on NAFLD or γ-GT were conducted mostly in male subjects. Second, to our knowledge, this is the first study to demonstrate individual and combined effects of γ-GT and diabetes on development of dementia in the general population. Moreover, our results clearly proved the link between γ-GT concentration and dementia risk, particularly EOD.

In conclusion, our data demonstrated that γ-GT is positively associated with future risk of dementia independent of diabetes and other metabolic conditions. The combined impact of γ-GT and diabetes increased risk for dementia development, implying that γ-GT concentration as well as glycemic status could be a risk factor. Understanding the role of γ-GT in the pathogenesis of dementia could enable clinicians to identify individuals most at risk, especially for EOD, with implications for prevention and treatment.

## Methods

### Study population

This longitudinal cohort study utilized data collected from participants in the National Health Insurance database maintained by the Korean National Health Insurance Service (NHIS), the single insurer in the Korean public health insurance sector that provides national health examinations for all Koreans. The NHIS database comprises the entire Korean population^23^; therefore, it can be used as a population-based, national source to study various diseases^24^. This biannual regular checkup includes anthropometric measurements, blood pressure, social habits, physical activity, and laboratory tests with overnight fasting. All biochemical samples are collected and measured as previously described^25^. Past medical history, alcohol consumption, smoking history, and exercise habits are collected by standardized self-reporting questionnaires. BMI is calculated as weight(kg)/height(m^2^) and obesity is defined as BMI ≥ 25 kg/m^2^, using the Asian-Pacific criteria^26^. All participants provided written informed consent to participate in the original NHIS. The study was approved by the institutional review board of the Yonsei University College of Medicine (4–2016–0575). All procedures performed in studies involving human participants were in accordance with the ethical standards of the 1964 Helsinki Declaration and its later amendments or comparable ethical standards.

### Definition of dementia and diabetes

To obtain information on incident dementia, we followed a cohort of 6,595,271 participants who were ≥40 years and were dementia-free NHIS beneficiaries (Fig. 1). Incident dementia was defined as having a diagnostic code of dementia (International Statistical Classification of Disease and Related Health Problems, 10th revision, [ICD-10] codes F00, G30, F01, F02, F03, G23.1, G31.0, G31.1, G31.82, G31.83, G31.88, and F10.7) with simultaneous prescription of an anti-dementia medication. Anti-dementia medications consisted of an acetylcholinesterase inhibitor (rivastigmine, galantamine, or aricept) or N-methyl-D-aspartate receptor antagonist (memantine). Patients with dementia were grouped into AD (ICD-10 codes F00, G30) or VaD (ICD-10 code F01) for subgroup analyses. The date of dementia diagnosis was the date when prescription of an anti-dementia medication and a dementia code coincided. The type of dementia was defined as the first diagnosis code of dementia. The index period was from January 1, 2007 to December 31, 2008. Participants were followed until the first diagnosis of dementia or until December 31, 2015. To determine the dose-response relationship between serum γ-GT and dementia incidence, we categorized sex-specific γ-GT by quartile or decile. In addition, we divided by age at the time of study recruitment (40–59, ≥60 years) to designate as EOD (40–59 years) and LOD ( ≥ 60 years)^27^. NAFLD was estimated by a calculating hepatic steatosis index (HSI) and defined by HSI ≥ 36^28^.

A diagnosis of diabetes based on ICD-10 codes included the principal diagnosis and up to four accompanying diagnoses. To investigate the effect of diabetes and γ-GT, we categorized subjects into 4 groups according to glycemic status: 1) Prevalent diabetes was defined as at least one service claim with a diagnosis of diabetes, either in outpatient or inpatient care and at least one prescription of a hypoglycemic agents during the index period; 2) Incident diabetes was defined as individuals without diabetes diagnosis or treatment, but whose fasting glucose concentration was ≥126 mg/dL; 3) IFG was defined as fasting glucose concentration ≥100 mg/dL and <126 mg/dL; and 4) Others were identified as normoglycemia. In addition, we defined subject who had diabetes diagnosis code and prescribed medication, but did not maintain (less than 3 years) the prescribed medication as treatment failure group whereas maintained the anti-diabetic medication prescription more than 3 years as treatment maintenance group.

Hypertension was defined as ICD-10 codes (I10–13, I15) plus treatment with anti-hypertensive agents, or systolic or diastolic blood pressure ≥140 mmHg/≥90 mmHg; dyslipidemia was ICD-10 code of E78 plus treatment with lipid-lowering agents or total cholesterol ≥240 mg/dL. A previous medical history of ischemic stroke or myocardial infarction was defined as ICD-10 codes of I63-I64 or I21-I22. We identified low social economic status as the lowest quartile for income in the study population^29^. Heavy alcohol consumption was defined as ≥ 30 g per day, and regular exercise was categorized as ≥3 times per week of moderate to vigorous physical activity.

### Statistical analysis

Descriptive characteristics were mainly presented as mean ± standard deviation (SD). As AST, ALT, and γ-GT were not normally distributed, those markers were expressed as median with interquartile range (IQR). As a sex difference in γ-GT level has been documented^30^, we categorized sex-specific cut-off. Data were expressed as numbers and as a frequency percentage. The χ^2^ test was used to determine differences in percentages of categorical variables, and the independent Student’s *t* test evaluated differences between the means of two continuous variables. A one-way analysis of variance (ANOVA) was used to compare the baseline characteristics of continuous variables by γ-GT categories. Incidence rates were expressed as events per 1,000 person-years, and were adjusted for age and sex using the direct method. Cox proportional hazards regression analysis was used to identify the association between γ-GT level and dementia after adjustment for other risk factors. The results were presented as HRs and 95% CI. Subgroup analysis was performed according to covariates including sex, age, hypertension, previous cardiovascular disease (ischemic stroke and myocardial infarction) history, obesity, and alcohol consumption. Dementia-free survival according to γ-GT categories and glycemic status was analyzed using the Kaplan-Meier curve and expressed as adjusted HR and 95% CI. A two-sided *P* value <0.05 was considered statistically significant. Statistical analyses were performed using SAS version 9.4 (SAS Institute, Cary, NC).

### Ethics approval and consent to participate

All participants provided written informed consent to participate in the original NHIS. The study was approved by the institutional review board of the Yonsei University College of Medicine (4–2016–0575).

## Data Availability

This study used the National Health Insurance Service NHIS 2009–2015 data, which were released by the KNHIS. Access to NHIS-NSC data are available from the website of NHIS (https:// nhiss.nhis.or.kr) after completing the application process and receiving approval (http://nhiss.nhis. or.kr/bd/ab/bdaba021eng.do).
